# Diverse Pathways of Engineered Nanoparticle-Induced NLRP3 Inflammasome Activation

**DOI:** 10.3390/nano12213908

**Published:** 2022-11-05

**Authors:** Xin Liao, Yudong Liu, Jiarong Zheng, Xinyuan Zhao, Li Cui, Shen Hu, Tian Xia, Shanshan Si

**Affiliations:** 1 Department of Dentistry, The First Affiliated Hospital, Sun Yat-Sen University, Guangzhou 510080, China; 2 Department of Endodontics, Stomatological Hospital, Southern Medical University, Guangzhou 510280, China; 3 Department of Oral and Maxillofacial Surgery, Stomatological Hospital, Southern Medical University, Guangzhou 510280, China; 4 School of Dentistry and California NanoSystems Institute, University of California, Los Angeles, CA 90095, USA; 5 Division of Nanomedicine, Department of Medicine, University of California, Los Angeles, CA 90095, USA; 6 Department of Oral Emergency, Stomatological Hospital, Southern Medical University, Guangzhou 510280, China

**Keywords:** engineered nanomaterials, NLRP3 inflammasome, property activity relationship, safer-by-design

## Abstract

With the rapid development of engineered nanomaterials (ENMs) in biomedical applications, their biocompatibility and cytotoxicity need to be evaluated properly. Recently, it has been demonstrated that inflammasome activation may be a vital contributing factor for the development of biological responses induced by ENMs. Among the inflammasome family, NLRP3 inflammasome has received the most attention because it directly interacts with ENMs to cause the inflammatory effects. However, the pathways that link ENMs to NLRP3 inflammasome have not been thoroughly summarized. Thus, we reviewed recent findings on the role of major ENMs properties in modulating NLRP3 inflammasome activation, both in vitro and in vivo, to provide a better understanding of the underlying mechanisms. In addition, the interactions between ENMs and NLRP3 inflammasome activation are summarized, which may advance our understanding of safer designs of nanomaterials and ENM-induced adverse health effects.

## 1. Introduction

In recent years, engineered nanomaterials (ENMs), as nanoscale materials synthesized with precise physicochemical properties in specific applications, have been applied in almost all fields; this includes optoelectronics, electronics, magnetism, medical imaging, drug delivery, cosmetics, sunscreens, catalysis, stain-resistant fabrics, dental bonding, construction, agriculture, corrosion resistance, and coating applications [1,2,3]. According to the Emerging Nanotechnology Project, three to four new consumer products containing nanotechnology are introduced to the market every week [4]. Since ENMs are widely used in our daily life, concerns about their effects on human health have been raised. With the common characteristics of small size and high surface-to-volume ratios, ENMs may have chemical, physical, and biological properties that are markedly different from fine particles with similar chemical composition [5]. Numerous nanotoxicological studies have shown that ENMs have the potential to elicit biological and toxicological responses, both in vitro and in vivo. For ENMs, the cell membrane cannot organize their entry when their size is less than 100 nm, the nucleus cannot prevent their entry when their size is approximately equal to 40 nm, and they can pass the blood-brain barrier when they are less than 35 nm. Smaller ENM can intensify the binding capacity, the potential to generate reactive oxygen species (ROS), the adsorption rate and catalytic activity, which may affect in vivo residence time [1,6]. A few nanomaterials, including carbon nanotubes, graphene and graphene oxide, titania, ceria, zinc oxide, nano silica, and nano silver, may affect the immune function [7]. A growing body of evidence has suggested that ENM exposure can, directly or indirectly, interact with cardiovascular (CV) components, leading to adverse events and worsening CV complications [1]. Some ENMs have been reported to cause acute lung inflammation, chronic pulmonary granuloma, and fibrosis [2].

**Figure 1:**
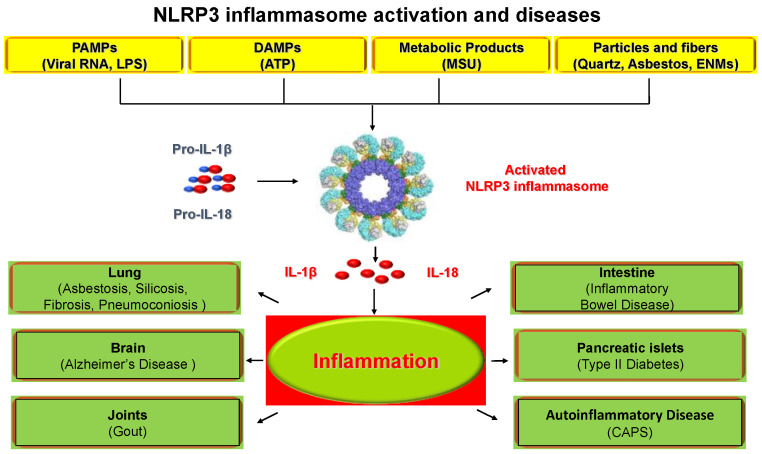
Stimuli for NLRP3 inflammasome activation and NLRP3 inflammasome-associated diseases. NLRP3 inflammasome is activated through a wide range of stimuli including pathogen-associated molecular patterns (PAMPs), damage-associated molecular patterns (DAMPs), metabolic products, and environmental hazards including engineered nanomaterials (ENMs). The activation of NLRP3 inflammasome and subsequent secretion of IL-1β and IL-18 have been associated with many diseases including lung fibrosis, asbestosis, silicosis, gout, Alzheimer’s disease, inflammatory bowel disease, type II diabetes, and cryopyrin-associated periodic syndrome (CAPS).

As a family of multi-protein complexes, inflammasomes are accepted as a major regulator of the host immune system upon exogenous stimuli [8]. A pattern recognition receptor (PRR) is required for inflammasome assembling as a sensor, and five PRRs, including NLRP1, NLRP3, NLRC4, AIM2, and Pyrin, have been verified to generate inflammasomes [9]. Additionally, other members of PRRs, such as NLRP2, NLRP6, NLRP7, NLRP12, and IFI16, were also reported to contribute to the formation of inflammasomes [10]. NLRP3 inflammasome is the most studied one among these inflammasomes, which contains a pyrin domain (PYD), a central nucleotide-binding domain (NBD), and a leucine-rich repeat (LRR) domain. NLRP3 has a vital impact on the innate immune system as it is crucial for activating caspase-1 as well as secreting interleukin-1β (IL-1β) and interleukin-18 (IL-18) [11]. PYD can interact with each other and then NLRP3 is activated to recruit the associated speck-like protein (ASC). The following caspase-1 activated by ASC induces bioactive IL-1β and IL-18, which affect cells in the immune system [12]. To date, the activation of the NLRP3 inflammasome has been attributed to several molecular and cellular events, including ionic flux, mitochondrial dysfunction, ROS generation, etc. [13]. In addition, some regulators responsible for NLRP3 inflammasome activation have been identified, such as guanylate-binding protein 5 (GBP5), double-stranded RNA-dependent protein kinase (PKR), and Nek7 [14,15,16,17]. As shown in Figure 1, it is now well-documented that the activation of NLRP3 inflammasome has detrimental health consequences, including atherosclerosis [18], gout [19], type II diabetes [20], silicosis [21], and lung fibrosis [22], etc. Furthermore, a series of studies have confirmed that NLRP3 inflammasome activation is linked to ENMs with various physicochemical properties, such as titanium oxide nanobelts [23], carbon nanotubes [22], and cerium oxide nanowires [24], as well as nanoparticles with certain surface modifications [25], indicating the crucial role of NLRP3 inflammasome in ENMs-induced biological responses. However, much work remains to be carried out to elucidate the mechanisms by which ENMs cause inflammasome activation.

In this article, we have discussed the diverse pathways of ENMs-induced NLRP3 inflammasome activation and summarized the physicochemical properties and activation mechanisms in major ENMs, including metal oxides such as rare earth oxide and V_2_O_5_, carbon nanotubes, nanowires with high aspect ratio, fumed silica, nanocellulose materials, and 2D ENMs including molybdenum disulfide, graphene, and graphene oxide. We have also reviewed the engineering approaches for less potential exposure toxicity of nanomaterials, which will exert a positive effect on developing sustainable nano-applications.

## 2. Mechanisms Involved in NLRP3 Inflammasome Activation by ENMs

### 2.1. Lysosomal Damage and Cathepsin B Release

One well-studied mechanism of NLRP3 activation is lysosomal damage caused by the phagocytosis of crystalline materials or proteinaceous aggregates. Although the exact mechanism of lysosomal injury is still unknown, cathepsin release from the lysosomal compartment is a necessary step for NLRP3 inflammasome activation [26]. However, cells lacking several cathepsins are still capable of activating this pathway, indicating that NLRP3 can be activated in a cathepsin-independent manner. Multiple cathepsins that CA-074-Me can block are involved in NLRP3 priming and activation [27]. The lysosomal protease cathepsin B, which is linked to lysosomal damage, has attracted the most attention. It has been established that cathepsin B directly interacted with NLRP3 at the endoplasmic reticulum in response to stimulation by a variety of NLRP3 activators, including ATP, nigericin, and particulate matter [28]. Furthermore, phagocytosis-related K^+^ efflux can be found at this time [29]. Rapid K^+^ efflux is a universal mechanism of NLRP3 inflammasome activation when exposure to Al(OH)_3_, silica, calcium pyrophosphate crystals, and Leu-Leu-O-methyl ester (a lysosomal damaging dipeptide). The mechanism underlying particulate matter-induced lysosomal rupture to K^+^ efflux remains unclear.

Mechanistically, lysosomal damage and cathepsin B release caused by ENMs activate the NLRP3 inflammasome. Hornung et al. demonstrated that lysosomal damage was required for the activation of the NLRP3 inflammasome, which was accompanied by the release of cathepsin B in the cytosol [26]. Intriguingly, as a result of reduced cathepsin B activity and suppressed phagosomal acidification, the lysosomal damage caused by solutions without the addition of crystalline silica and aluminum salt stimuli also triggered NLRP3 inflammasome activation. In addition, nanodiamonds were capable of rupturing the lysosomal membrane, which subsequently induced cathepsin B release and NLRP3 activation [30]. The release of lysosomal cathepsin B was observed in conjunction with the IL-1β secretion induced by graphene oxide (GO) [31]. Small GO, as opposed to large GO, caused a more efficient release of cathepsin B from the lysosomal compartment. The higher charge density of small GO sheets caused them to be more hydrophilic than the larger ones [32]. A recent study found that high doses of GO (200 g/mL for 6 h) may disrupt the plasma membrane, and nanosized graphene sheets extracted phospholipids from membranes in a mammalian cell model [33]. Therefore, it is rational to hypothesize that compared to small GO, large GO sheets within lysosomes have less interaction with the lipids from the membrane, which leads to less lysosomal damage and cathepsin B release.

Carbon nanotubes (CNTs) represent one of the extensively researched nanomaterials that activate the NLRP3 inflammasome. After treating human monocyte-derived macrophages (MDM) with crocidolite asbestos or tangled or rigid, long multi-walled CNT (MWCNT) for 6 h. Bioinformatics analysis was performed to explore the expression patterns of secreted proteins, and lysosomal protein was found to be secreted following material exposure [34]. In accordance with the above findings, Svadlakova et al. showed that MWCNT and graphene platelets activated the NLRP3 inflammasome via lysosomal instability and cathepsin B release [35].

The different sizes and shapes of CNTs exert different effects on NLRP3 inflammasome activation, and the diameter and rigidity of CNTs are also responsible for their bioreactivities. Palomaki et al. showed that needle-like CNTs triggered lysosome damage, cathepsin B release, NLRP3 inflammasome stimulation and IL-α and IL-1β production [36]. Thin, needle-like MWCNTs (diameter 50 nm) have also been shown to give rise to stronger inflammation and cytotoxicity than thick MWCNTs (diameter 150 nm) of similar lengths [37]. However, Nel et al. did not consider the strongly aggregated, needle-like CNTs with a diameter of >30 nm as typical CNTs. Instead, the material characteristics (length, diameter, and aspect ratio), suspension state, and surface properties may become the critical factors of CNT-induced NLRP3 inflammasome activation for typical CNTs with a diameter of less than 30 nm [38]. An MWCNT library created by Wang et al. includes as-purified (AP), purified (PD), and carboxylated (COOH) forms [39]. When compared to non-dispersed tubes, bovine serum albumin (BSA)-dispersed tubes were found to cause more lysosomal impairment, cathepsin B release, NLRP3 inflammasome activation, and IL-1β production in mice. In addition, pluronic F108 (PF108) coating has the potential to reduce tube surface bioactivity, lysosomal damage and cathepsin B release, which provides new insight into the inhibition of NLRP3 inflammasome activation and the reduction of pro-inflammatory cytokines [22].

A mechanistic insight of how rare earth nanoparticles (REOs) cause lysosomal destruction was attained by Li et al. They illustrated that RE ions (III) shedding in acidifying macrophage lysosomes resulted in the crystallization of REPO4 sediments on the particle surfaces. Once the free lysosomal phosphates were exhausted, nanoparticles biotransformed into urchin-shaped or mesh-like structures by stripping phosphate groups from phospholipids on the lysosomal membrane, triggering a chain of events including lysosomal damage, NLRP3 inflammasome activation, and IL-1β release. This series of reactions led to the production of profibrogenic cytokine TGF-β1 and PDGF-AA in lung epithelial cells, resulting in pulmonary fibrosis. The biological transformation and pro-fibrogenic effects were, however, blocked by pretreating REO nanoparticles with phosphate in a neutral pH environment to form a protective coating layer. This serves as a safer design strategy (surface coating) for creating REOs in biological applications [40]. Furthermore, Mirshafiee et al. found that while REO nanoparticles co-localized with the LAMP-1 positive compartment (lysosomes) in both KUP5 and Hepa 1-6 cells, only KUP5 cells exhibited lysosomal injury, which in turn recruited caspase 1 and activated the NLRP3 inflammasome [41]. The observed difference is due to the differential pH levels in lysosomes between Kupffer cells and hepatocytes.

Ji et al. created a library of ceria nanomaterials to investigate the effects of lengths and aspect ratios on ENMs-induced NLRP3 inflammasome activation. A critical length and aspect ratio of CeO_2_ nanorods may affect the lysosome destabilization and cathepsin B release [24]. The findings demonstrated that CeO_2_ nanorods triggered less lysosomal destabilization, cathepsin B release, and IL-1β production when their length was less than 200 nm and aspect ratio was below 22. Scanning electron microscope images displayed micron-sized stacking bundles that were capable of penetrating cell membranes. Lysosomes with a diameter of 1.1–2.9 μm were pierced by the long stacking bundles. In addition, Hamilton et al. showed that the short and spherical nanobelts did not promote lysosomal damage and cathepsin B release, whereas the long fiber-shaped titanium dioxide nanobelts did, indicating length-dependent bioactivity should be considered to create nanoproducts [23].

### 2.2. TLR4 and NF-κB Activation

Toll-like receptors (TLRs) are essential in the innate immune system by binding to ligands and then activating transcription factors, NF-κB and activator protein-1 (AP-1), stimulating the expression of inflammatory genes pro-IL-1β and NLRP3, which are essential for activation of the NLRP3 inflammasome. TLRs play a key role between cells and nanomaterials [42]. NF-κB is a crucial nuclear transcription factor associated with a variety of cellular activities such as stress response, apoptosis, and inflammatory regulation [43]. It was demonstrated that TiO_2_ nanoparticles (NPs) induced neuroinflammation in the mouse hippocampus, TLRs were involved in the associated inflammatory response after the uptake of TiO_2_ NPs. TiO_2_ NPs promoted TLR4 expression, and increased TLR4 expression caused the activation of NF-κB-inducible kinase (NIK), which subsequently produced phosphorylation of IKKs and IκB, ubiquitination and degradation of IkB, and finally caused activation of NF-κB [42].

Large GO (L-GO from 500–2000 nm) induced ROS production and TLR-4 activation, promoted macrophage polarization and the secretion of the pro-inflammatory cytokines IL-1β and TNF-α, and additionally activated the NF-κB signaling pathway, which ultimately initiated IL-6 expression in hepatocytes [44]. The exposure of CuO NPs caused J774A.1 macrophages to express pro-IL-1β and subsequently activated NF-κB via the MyD88-dependent TLR4 signaling pathway. The major bridging protein downstream of TLRs contains MyD88, which has a role in causing the expression of pro-inflammatory genes and activating the NF-κB pathway [45]. Amorphous silica NPs accelerated the translocation HMGB1 from the nucleus to the cytoplasm and its extracellular release by inducing lysine acetylation of HMGB1. Extracellular HMGB1 then was recognized and bonded to TLR4 on target cells, activating the MyD88 and NF-κB signaling pathways, and ultimately triggering cellular inflammation [46]. Rats were administered with mesoporous silica nanoparticles (MSNs) at different concentrations for 30 days, and the activation of TLR4/MyD88/NF-κB pathway was found in MSNs-treated rat samples [47].

### 2.3. ROS Generation

Under physiological conditions, ROS is essential in cellular signal transduction responding to stress [48]. The cellular antioxidant system will be overloaded if the ROS generation is sustained, leading to oxidative stress, cell injury, and potentially cell death. The activation of the NLRP3 inflammasome caused by ENMs has also been linked to ROS production.

Shirasuna et al. reported that nano silica (NS) particles generated ROS, increased NLRP3 inflammasome activity, and placental inflammation, leading to the pregnancy problems in female mice. In addition, the suppression of ROS production entirely shielded against pregnancy complications induced by NS [49]. ROS played a crucial role in the NLRP3 inflammasome activation, IL-1β production, and macrophage-guided myofibroblast transformation caused by NS and CNTs [50]. Due to the sophisticated physicochemical modification, substantial pore volume, and high specific interface area, MSNs are ideal to serve as a drug carrier [51,52,53]. New evidence on the processes behind MSN hepatotoxicity was revealed by Zhang et al. The activation of NLRP3 inflammasomes, triggered by MSN-induced ROS production, was postulated to be the mechanism by which MSNs promoted liver inflammation and hepatocyte pyroptosis [54]. Deng et al. showed that, due to MSN exposure, ROS generation in Caco-2 cells was enhanced with increasing MSN concentration and subsequently activated the NLRP3 inflammasome. In addition, intestinal inflammation was exacerbated by the overactivation of the NLRP3 inflammasome [55].

Inflammation is caused by exposure to polystyrene nanoparticles (PSNPs). Chi et al. found that exposure to PSNP significantly increased the levels of neutrophil extracellular traps (NETs) and local neutrophil infiltration in the liver. PSNP exposure ruptured the cell membranes of AML12 cells cultivated in vitro, producing a significant quantity of ROS and activating the NLRP3 inflammasome pathway. Further investigation indicated a strong relevance between the ROS-NLRP3 axis and PSNP-induced NET formation. The NET formation induced by PSNPs in AML12 cells could be attenuated by both ROS and NLRP3 inhibition. In turn, cultivated with deoxyribonuclease I (DNase I)-coated PSNPs dismantled NET and impeded the formation of ROS-NLRP3 axis in mice [56]. In a PSNPs exposure or PSNPs/LPS co-exposure mouse model, He et al. revealed that exposure to PSNPs enhanced permeability and induced duodenal inflammation. PSNPs had the potential to increase oxidative stress and ROS generation, stimulate the NF-κB/NLRP3 pathway, and promote the expression of pro-inflammatory factors such as TNF-α, IL-6, and IFN-γ [57].

Winter et al. demonstrated that TiO_2_ nanoparticles induced the NLRP3 inflammasome via ROS signaling in murine bone marrow-derived dendritic cells (BMDCs) [58]. In a mouse model of allergic pulmonary illness caused by ovalbumin, TiO2 NPs elevated ROS levels and promoted the expression of IL-1β, IL-18, and inflammasome-associated proteins, exacerbating airway inflammation and hyperreactivity [59].

Carbon black nanoparticles (CBNPs) were able to produce ROS, which in turn decreased miR-96 expression and, therefore, upregulated FOXO3a expression. The increased FOXO3a subsequently engaged in the activation of NLRP3 inflammasome [60]. Zhu et al. reported that in comparison to other groups of nanoparticles in sizes, ultrasmall (4.5 nm) Au nanoparticles (Au4.5) generated the highest amount of intracellular ROS, which was promoted by cell cytoplasm penetration and targeted autophagy protein-LC3 (microtubule-associated protein 1-light chain 3) for degradation [61].

Since ROS is mainly produced by NADPH oxidase and mitochondria in macrophages, there is an increasing amount of study on their further links with NLRP3 inflammasome activation. The transmembrane enzyme complex known as NADPH oxidase consists of membrane components (gp91^phox^ and p22^phox^) and cytosolic components (p67^phox^, p47^phox^, p40^phox^, and Rac-GTP binding protein) [62]. When p67^phox^, p47^phox^, and p40^phox^ are phosphorylated, NADPH oxidase is activated, promoting the reduction of molecular oxygen into superoxide. The precursor of hydrogen peroxide is then further catalyzed to produce ROS [63]. Sun et al. showed that NADPH oxidase played a crucial role in activating NLRP3 inflammasome and lung fibrosis. CNTs and silver nanowires enhanced lysosomal damage, oxidative stress, and subsequent NLRP3 inflammasome activation through the NADPH oxidase pathway. These responses were attenuated in p22^phox^ deficient THP-1 cells and p47^phox^ deficient bone marrow-derived macrophages (BMDMs). In addition, p47^phox^ deficient mice did not develop lung fibrosis induced by MWCNTs [64]. Another study evaluated ROS factors for iron oxide nanoparticles (IONPs)-induced inflammasome activation. The octapod and plate IONPs generated much more ROS than the cube and sphere IONPs. Inhibiting NADPH oxidase dramatically reduced the IL-1β generation induced by IONPs [65], and it appeared that ROS produced from NADPH oxidase was essential for the inflammasome-activating characteristic of IONPs. Additionally, inflammasome response and oxidative damage might be triggered in cardiomyocytes by silica nanoparticles (SiNPs). The in vivo data showed that SiNPs caused histological damage and ultrastructural alteration in heart tissue, as well as the occurrence of oxidative damage and elevated amounts of inflammatory factors (IL-18 and IL-1β). SiNPs increased the production of intracellular ROS in vitro and stimulated the NLRP3/Caspase-1/GSDMD signaling pathway in cardiomyocytes. In contrast, the NADPH oxidase inhibitor substantially reduced the amount of ROS and decreased the production of NLRP3, IL-18, and cleaved-IL-1β [66].

Certain ENMs may directly affect mitochondria. The rupture of the mitochondrial membrane, loss of the mitochondrial membrane potential, and subsequent formation of ROS might all be the results of the ENMs-mitochondria interaction. Once mitochondria overproduce ROS, the lysosome membrane may become unstable and release cathepsin B, which in turn activates caspase-2 to promote mitochondrial permeability and cytochrome c release [67]. Additionally, cathepsin D, another released lysosomal protease, can activate Bax to become a contributing factor in mitochondrial rupture and cytochrome c release [68]. The mitochondrial production of ROS may increase as a result of cytochrome c release [69]. These results imply that mitochondria-induced ROS might create a positive feedback loop that will increase the generation of ROS. 

Liang et al. found that zinc oxide nanoparticles (ZnO-NPs) exposure led to ROS production in A549 cells. After ZnO-NPs treatment, there was a considerable rise in the expression of NLRP3 as well as the release of IL-1β and IL-18, proving that ZnO-NPs had activated the NLRP3 inflammasome. The ROS scavenger prevented the NLRP3 inflammasome from being activated by ZnO-NPs in the meantime [70]. Chen et al. also investigated the probable causes of skin injury brought on by the combination of ZnO-NPs with ultraviolet radiation (UVB). They showed that ZnO-NPs and UVB caused significant mitochondrial dysfunction, including a decrease in mitochondrial membrane property and mtROS generation, which activated the NLRP3 inflammasome in keratinocytes [71]. It was demonstrated that Ag, CuO, and ZnO nanoparticles induced caspase3-related apoptosis, along with ion shedding and generation of mtROS in Kupffer cells (KCs) and hepatocyte cell lines [72]. For KCs, Li et al. found that GOs were phagocytized, which induced plasma membrane lipid denaturation, resulting in mtROS burst, and NLRP3 inflammasome stimulation [73]. In addition, Sunasee et al. recently discovered that a stinger shape cationic derivative (cellulose nanocrystals-grafted poly-N-aminoethylmethacrylamide, CNC-AEMA2) increased the level of NLRP3 inflammasome-dependent IL-1β in mouse and human macrophages, which was associated with the significant mtROS generation [74]. Feng et al. showed that exposure to Nano-Co triggered mtROS generation and NLRP3 inflammasome activation in hepatocytes. Nano-Co formed NLRP3 inflammasome after oxidative stress initiation, suggesting a prominent influence of mtROS-NLRP3 interaction in hepatotoxicity induced by Nano-Co [75].

Due to the distinctive photophysical properties, such as high brightness, size-tunable fluorescence, excellent photostability, and broad absorption combined with narrow emission, quantum dots (QDs), which belong to semiconductor nanocrystals, are one of the most promising nanoparticles for biological and biomedical applications [76]. In hepatic L02 cells, CdSe/ZnS QDs elevated the production of mtROS, and the NLRP3 activation caused by QDs was reduced by a mtROS scavenger [77]. The QDs also induced NLRP3 activation in liver tissue in vivo. Additionally, following the administration of QDs, an increase in mtROS was observed in the liver, and the mtROS scavenger inhibited the activation of NLRP3. These findings indicate that QDs generate mtROS, which leads to NLRP3 inflammasome activation, hepatocyte pyroptosis, hepatic inflammatory status, and liver impairment. As a component of low-toxic chemicals, silver selenide (Ag_2_Se) QDs provide promising possibilities for the use of QDs in the biomedical area. However, it was shown that cadmium-containing QDs in the hippocampus caused significant inflammatory reactions which were harmful to the central nervous system. Ag_2_Se QD-induced ROS production, especially mtROS, activated the NLRP3 inflammasome and induced caspase-1 to convert pro-IL-1β into functional IL-1β release [78].

Nanoparticles with surface coating and functionalization can potentially have an impact on ROS production. Lunov et al. demonstrated that the oxidation of the redox-active thioredoxin (TXN) and its subsequent separation from thioredoxin-interacting protein (TXNIP) might result from mitochondrial dysfunction and ensuing ROS generation caused by amino-modified polystyrene nanoparticles. The released TXNIP may interact with NLRP3 and cause a conformational shift, which in turn caused NLRP3 to bind adaptor protein ASC, activating the inflammasome and releasing IL-1β [79]. In addition, it was shown that ROS generation, NLRP3 inflammasome activation, and IL-1β production were induced by unmodified microsized 1000-nm amorphous silica particles (mSP1000). Morishige et al. examined the levels of IL-1β generated by THP-1 cell exposure to functionalized mSP1000s with a polar group (-COOH, -NH_2_, -SO_3_H, -CHO). The surface functionalization of mSP1000 significantly inhibited IL-1β level by decreasing ROS generation, even though unfunctionalized and surface-functionalized mSP1000s were phagocytosed into the THP-1 cells with similar frequencies [25]. A lanthanide nanocrystals (LNs)-specific surface coating peptide called RE-1 was previously discovered by Zhang et al., which inhibited LNs-induced autophagy and cytotoxicity in liver hepatocytes and HeLa cells [80]. Yao et al. showed that RE-1 coating dramatically suppressed LNs-induced inflammatory response, NLRP3 inflammasome activation, and intracellular ROS in vitro and in vivo [81]. 

### 2.4. Plasma Membrane Perturbation

Studies have shown that molecular interactions of NPs with their target cells play a key role in regulating membrane potential and associated downstream intracellular events that may cause disruption of membrane integrity, leading to abnormalities in the actin network and affect ion exchange [82]. The clustering of NPs into specific sizes and shapes enhances their ability to deform the membrane and produce invaginations and intraluminal vesicles. The formation of membrane invaginations and intraluminal vesicles, along with the pressure generated by AgNPs attached to the membrane, increases membrane tension and may induce membrane perforation [83].

The physiological and biochemical functions of cells depend on their proper membrane fluidity. Nano silica at 20 nm was found to induce a significant increase in ROS production and intracellular Ca^2+^ in macrophages, while also resulting in a decrease in membrane fluidity. The elevated ROS and free intracellular calcium were involved in the perturbation of membrane integrity and intracellular calcium homeostasis, respectively [84]. Cell membranes play a crucial role in maintaining the dynamic balance of the intracellular environment and regulating the transport of substances. The membrane perception of membrane perturbation by NLRP3 inflammasomes is caused by the interaction between the silanol surface of fumed silica aggregates and the cell membrane, which subsequently stimulates the production of cytokine IL-1β. Fumed silica activated NLRP3 inflammasome through a non-lysosomal mechanism, and it damaged the integrity of cell membranes and stimulated the production of ROS, both of which could cause inflammasome activation [85,86]. Recent studies showed that fumed silica nanoparticles were no longer safe due to their chain-like structure and the high trisiloxane ring, as well as surface silanol density on the particle surface. The chain structure prevents silica from being absorbed by the cells, and the cell membranes adsorb the particles. The surface of the trisiloxane rings is readily hydrolyzed to form hydrogen-bonded silanol groups (e.g., ≡Si-OH) that are capable of generating large amounts of ROS, leading to cell membrane perturbation, potassium efflux, and eventual activation of NLRP3 and IL-1β. [87].

In a recent study, amorphous aluminum hydroxyphosphate nanoparticles (AAHPs) were found to initiate interactions with cells in a surface charge-dependent manner, and positively- charged (Posi) caused the most significant cell membrane perturbation, which induced potassium efflux in BMDMs. In addition, Posi showed the highest level of depolarization and triggered more Posi leakage than other types of charged AAHPs. AAHPs induced plasma membrane perturbations accompanied by ion permeability. Potassium efflux triggered cellular responses which enhanced the pro-inflammatory effects of the corresponding particles. Moreover, the involvement of the NLRP3 inflammasome was confirmed in experiments with aluminum nanophosphate and NLRP3 gene-deficient THP-1 cells. In addition, AAHPs induced IL-6 and IL-1β production in BMDCs. It is important to note that AAHPs promoted lysosomal damage, mitochondrial disability, and ROS production after being phagocytized [88].

### 2.5. Potassium Efflux

Potassium homeostasis is an important regulator of apoptosis, and cytosolic potassium efflux involved in the apoptotic cascade response, with related response enzymes including caspase, cytochrome c, and nucleic acid endonuclease [89]. Cellular potassium efflux, which can be activated by asbestos, monosodium urate, silica, cholesterol, and calcium phosphate crystals, is a mechanism that activates NLRP3 [90]. Polyethylene glycol-modified NPs increase potassium efflux, which readily escapes lysosomes, affects mitochondria, and induces mitochondrial apoptosis. Potassium efflux promotes the binding of caspase-9, AFP-1, and cytochrome c, inducing apoptosis. In addition, potassium ion efflux may lead to calcium ion inward flow, resulting in disturbed calcium ion concentration, and triggering apoptosis [91]. Fumed silica NPs have a strong correlation with potassium ion efflux, while both can generate oxygen radicals to induce additional cellular responses and enhance the pro-inflammatory effect of nanoparticles. NLRP3 inflammasomes are linked to the varied ENMs-induced IL-β production. In response to cytoplasmic matrix or plasma membrane-derived stimuli, ENMs aggregate the NLRP3, ASC, and caspase-1 subunits of inflammasomes [92]. Membrane mechanical stress can activate NLRP3 inflammasomes through mechanosensitive channel-mediated K^+^ efflux, and TiO_2_ nanospikes apply mechanical pressure to cells, leading to potassium efflux and the activation of inflammasomes in a caspase-1 and NLRP3-dependent manner in macrophages and dendritic cells during cellular uptake [93]. Higher extracellular K^+^ concentrations prevented fullerenol nanoparticles-induced IL-1β secretion from primary macrophages. This result suggests that, similar to other stimuli, K^+^ effluents during fullerenol nanoparticles exposure may also be a common trigger for the activation of NLRP3 inflammasome [94].

**Figure 2:**
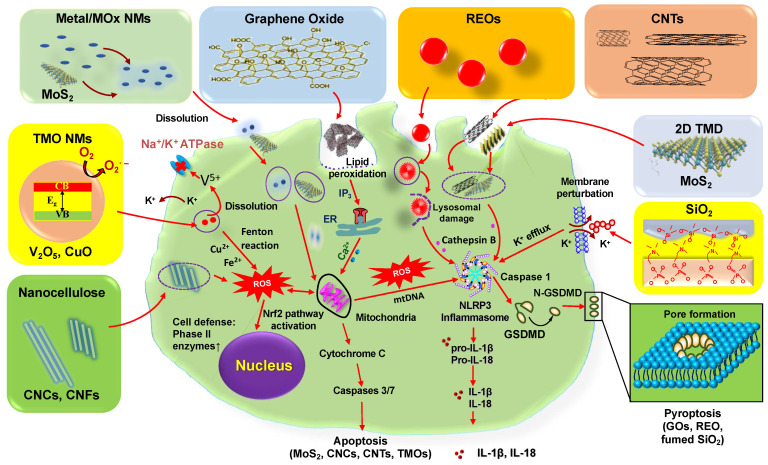
ENMs-induced NLRP3 inflammasome activation in macrophages. This includes the metal or metal oxide (MOx) or transition-metal oxide (TMO) NMs; for example, CuO induces NLRP3 inflammasome activation and apoptosis due to their dissolution and shedding of toxic ions and oxidative stress; REOs (e.g., Gd_2_O_3_, La_2_O_3_, and Y_2_O_3_) and GOs induce NLRP3 inflammasome dependent pyroptosis in macrophages. For REOs, the transformation from sphere to sea urchin-shaped and the formation of rare-earth phosphate (REPO_4_) structures on the lysosomal membrane, where RE(III) ions strip phosphate from the phospholipids, induce lysosomal damage, cathepsin B release, leading to NLRP3 inflammasome activation and GSDMD-mediated pyroptosis; the phagocytized GOs-induced NADPH oxidase activation leads to lipid peroxidation, triggering PLC activation that leads to calcium flux, mitochondrial ROS generation, and NLRP3 inflammasome activation, resulting in IL-1β production as well as subsequent pyroptosis. For fumed SiO_2_, the activation of NLRP3 inflammasome is involved in the pathway premised on K^+^ efflux resulting from the plasma membrane perturbation after SiO_2_ binding. Moreover, 2D transition metal dichalcogenides (TMD), CNCs, and CNTs induce ROS-mediated apoptosis and NLRP3 inflammasome-mediated inflammatory responses in macrophages after their internalization. V_2_O_5_ sheds V^5+^ ions that inhibit Na^+^/K^+^ ATPase, leading to K^+^ release from constitutive K^+^ channels, which activates NLRP3 inflammasomes.

A summary of molecular/cellular mechanisms underlying ENMs-induced NLRP3 inflammasome activation is shown in Table 1 and Figure 2.

## 3. Property Activity Relationships for ENMs

### 3.1. Surface Reactivity of Carbon Nanotubes (CNTs)

Engineered carbonaceous nanomaterials (ECNs) include single-wall carbon nanotubes (SWCNTs), multiwall carbon nanotubes (MWCNTs), graphene, and graphene oxides (GO). Due to the distinctive flexibility, high conductivity, surface strength, hydrophilicity, and dispersibility, there has been a steep rise in ECN applications in optoelectronics, catalysts, and drug delivery [38,95].

It has been demonstrated that ECNs can cause both acute and chronic lung damage. After intratracheal instilling, aerosolized inhaling, or oropharyngeal aspiring SWCNTs and MWCNTs, various inflammatory diseases were observed in the lungs of animal models [96,97,98,99]. During the progression of fibrosis, myofibroblasts are crucial for maintaining collagen synthesis and matrix contraction. Fibrogenic CNTs and silica elicit macrophage-guided myofibroblast transformation by elevating ROS generation, NLRP3 inflammasome activation, and IL-1β release in macrophages [50]. Graphene and GO have also been demonstrated to increase the risk of pulmonary inflammation and fibrosis [99].

Increasing evidence has demonstrated that the tube length, suspension state, charges, and surface coating are the main factors of NLRP3 inflammasome activation induced by CNTs [100,101]. While short and/or tangled MWCNTs were non-cytotoxic, long and rigid MWCNTs caused pyroptosis in macrophages, which increased the expression of NLRP3, Casp-1 and IL-1β. However, this effect did not occur in neutrophils [102]. Three short MWCNTs (length (1–2), (1–5), and (5–20) μm) and two long MWCNTs (NTlong1 and NTlong2, mean length 13 and 36 μm, respectively) were utilized to treat THP-1 macrophages. The results largely demonstrated length-dependent variations, even though the diameters of these tubes also differed from each other. Only the tubes NTlong1 and NTlong2 significantly increased the levels of IL-1β and IL-6, whereas NTlong1 by itself elevated IL-8 expression. Additionally, the production of cytokines in Met5a mesothelial cells was only considerably increased by the substances released by NTlong1 and NTlong2 treated macrophages [103]. Long and rigid MWCNTs with a needle-like appearance or appearing as individual fibers always displayed increased cytotoxicity and elicited the most IL-1β release in PMA-primed macrophages [104]. In an LDH release experiment, the long, needle-like MWCNTs showed higher bioactivity, increased IL-1α and IL-1β levels, and stimulated the generation of lysosomal and inflammatory proteins in human macrophages [34]. Similarly, Palomäki et al. explored the relationship between the length of MWCNT and biological activity [36], showing that bioactivity increased with the length.

It has also been revealed that MWCNTs with good dispersal abilities caused an increase in IL-1β production in THP-1 cells [39]. MWCNT dispersion may be a significant element that further affects cellular uptake. As opposed to neutral group modification, polar group functionalization broadens the dispersion of MWCNTs and promotes MWCNT further interaction with cells [105,106,107]. Both alterations reduce the probability of “frustrated phagocytosis”, which can emerge when cells find it difficult to take up the massive agglomerates generated by pure MWCNTs, and thus disrupt the cellular biological process triggered by the modified MWCNTs. The bio-effects of polar group-functionalized MWCNT fibers are enhanced in a concentration-dependent manner by a higher absorption rate, which also suggests an elevated intracellular concentration of these fibers [108]. The impact of modification groups themselves cannot be entirely ruled out as a result of their prevalence in biochemical molecules and the necessity of their participation in intracellular biological activities. Due to bare carbon structures, pure MWCNTs are not hydrophilic and aggregate into large structures in aqueous solutions, which affects their bioactivity, interface reaction, and potentially hazardous effects [109]. MWCNTs are commonly dispersed by dispersants in solutions for biomedical applications, including pluronic copolymer, dipalmitoylphosphatidylcholine (DPPC), and BSA. Profibrotic cytokines and IL-1β production were shown to be affected by the dispersion of AP, PD, and COOH MWCNTs by DPPC and BSA in vitro and in vivo [22].

Sager et al. created a mouse model with the administration of unmodified and MWCNT that were surface functionalized with -COOH. The results showed that MWCNT modified with the -COOH group significantly diminished the NLRP3 inflammasome activation and pulmonary toxicity [110]. The ability of endotoxin-free preparations of raw CNTs to trigger inflammatory reactions was shown to be relatively limited in vitro and in vivo [111]. Raw CNTs were purified and subjected to selective oxidation, which increased their dispersibility in physiological fluids, but also increased their inflammatory activity. This phenomenon is observed in spherical carbon nano-onions (CNOs) with a size of 6 nm. Additionally, CNO generated less inflammation than carbon nanotubes. On the contrary, the inflammasome activation of pure CNTs and CNOs are dramatically reduced as a result of the benzoic acid functionalization. An MWCNT library was created by Wang and Li et al. and contained MWCNTs that are AP, PD and modified with various functionalizations and charges, such as COOH, polyethylene glycol (PEG), amine (NH_2_), sidewall amine (sw-NH_2_), and polyetherimide (PEI) [39,112]. These nanotubes activate the NLRP3 inflammasome and produce IL-1β in the following order: COOH- or PEG<< NH2 or sw-NH2< PD <AP<< PEI-, which reflects the variations in purity, surface reactivity, and polarity. In BEAS-2B and THP-1 cell lines, the cationic PEI-functionalized tubes enhanced IL-1β, TGF-1, and PDGF-AA production related to inflammatory fibrosis, whereas neutral and weak cationic functionalization (NH_2_ and sw-NH_2_) exhibited intermediary effects. Conversely, anionic functionalization (COOH- and PEG-) reduced the biological effects. The different cellular uptake rates and the activated NLRP3 inflammasome caused by nanotubes with various charges are responsible for these discrepancies. Strongly positive charged particles tend to easily penetrate the cell membrane and enter the cell; therefore, they tend to cause prolonged retention time in cells and result in significant toxicity as a consequence of lysosomal disruption, NLRP3 inflammasome activation, and IL-1β generation [112,113].

After being taken up by cells, CNTs could cause lysosomal damage. As a result of the significant dispersion effects between the carbon structure and the lipid molecules, it has been hypothesized that the hydrophobicity of the pure bare carbon surface of CNTs enables a large number of phospholipids to dissociate from cell membranes [114]. Additionally, CNT surface flaws and functional groups may cause the generation of abiotic ROS. The capacity of CNT to cause lysosomal damage is due to their surface reactivity. Compared with unfunctionalized nanotubes, Al_2_O_3_-coated MWCNTs synthesized via atomic layer deposition increased IL-1β secretion, but decreased IL-6, TNF-α, and osteopontin production in THP1 cells and primary human macrophages [115]. According to research by Kim et al. [116], acid-treated MWCNT significantly reduced lung inflammation, while untreated MWCNT exerted the opposite effect. When compared to unmodified MWCNT, coating MWCNT with polystyrene considerably reduced pulmonary cytotoxicity and inflammation [105]. A tri-block copolymer coating PF108, composed of an interleaved hydrophobic poly propylene oxide domain and two hydrophilic polyethylene oxide chains, might prevent CNTs from exhibiting inflammatory impact [117]. The protective “brush-like” layer from PF108 could passivate the surface of the tube, hinder tube aggregation and disrupt interactions with the lysosomal membrane, which results in less cathepsin B release, NLRP3 inflammasome assembly, and IL-1β generation in macrophages. Thus, it is reasonable to consider that the PF108 coating is a promising strategy for the safer design of CNTs in nano-applications [98]. It is believed that substances coated on the MWCNT surface bestow the nanotube with particular physicochemical properties [118] and can alter or even cover the original effects of the MWCNT [115], which will be restored after clearing the coating material [119]. Collectively, these findings show that functionalizing or coating MWCNT surfaces with certain chemicals without changing their intrinsic structure may be a secure design strategy for reducing MWCNT toxicity when taking into account their potential usage in biomedical applications.

Other ECNs, such as SWCNTs and graphene, have also been demonstrated to have a similar link to the inflammasome. AP- SWCNTs were prepared after using the Hipco, arc discharge, and Co-Mo catalyst procedures. To enhance dispersion and colloidal stability, SWCNTs were then further purified or coated with PF108 or BSA. The pristine graphene samples were coated with BSA and PF108 to increase dispersion in the aqueous solution, and GO was synthesized in a small lateral size (GO- S) and a large one (GO- L) [98]. Regardless of the method of synthesis, AP- and PD-SWCNTs, as well as graphene (BSA) and GO (S and L), could induce the production of IL-1β and TGF-β1 in THP-1 and BEAS-2B cells. It was shown that the reactive surface, not metal impurities or other elements of SWCNTs, graphene, or GO, is the key physicochemical factor in activating inflammasome. 

Collectively, the above findings suggest that the size, dispersal state, and surface coating of ECNs are strongly related to the NLRP3 inflammasome activation. A series of well-orchestrated designs are needed to temper inflammatory potential before clinical applications.

### 3.2. Lateral Size of Graphene Oxide (GO)

**Figure 3:**
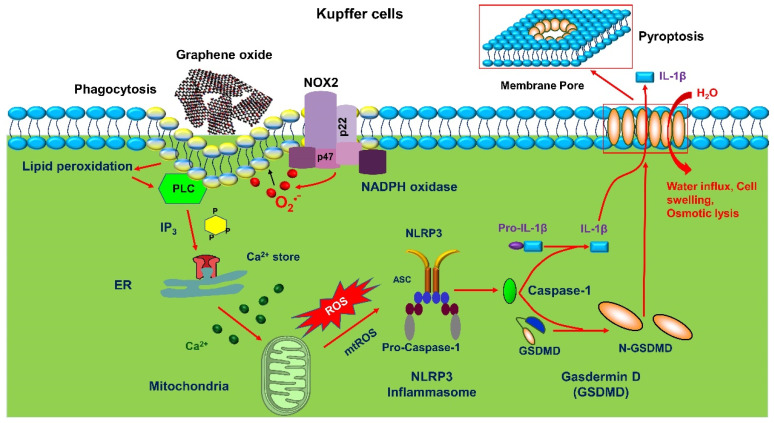
The cellular mechanism of GO-induced inflammatory effects. GOs were taken up into Kupffer cells through phagocytosis. Phagocytosis triggered NADPH oxidase mediated plasma membrane lipid peroxidation, leading to PLC activation, calcium accumulation, mtROS generation, caspase 1 activation as well as IL-1β release and subsequent GSDMD-mediated pyroptosis.

GO is a representative two-dimensional material composed of a simple layer of carbon atoms arranged mainly in a regular hexagonal shape. Due to their unique geometric structure, GOs exhibit outstanding properties such as huge surface area, high flexibility, good dispersion in aqueous solutions, hydrophilicity, and excellent biocompatibility [73,120]. Additionally, GOs have been developed in the last decade for electronics, optics, sensors, and filtration, and owing to the ability of properties modulation, graphene derivatives have been used in a wide range of fields, including applications related to drug delivery systems, gene therapy and contrast agents [121]. There have been various studies on the effect of GO lateral size in recent years. For example, graphene quantum dots (GQDs) (an ultra-small GO with lateral size < 100 nm) with low cytotoxicity and size consistency suggest that they can be used as carriers for targeted drug delivery [122]. The effects of micron-sized GO on peritoneal macrophage and murine macrophage J774A.1 cell line, murine Lewis lung cancer, human breast cancer, human hepatoma cell, and human umbilical vein endothelial cell were observed. A stronger inflammatory response was induced, while the nano-sized graphene sheets showed better biocompatibility [123]. It was reported that hepatocytes, LSECs, and Kupffer cells have different cell membrane uptake of GO with different lateral sizes. GO uptake by phagocytosis induced a series of cascade reactions including NADPH oxidase-mediated plasma membrane lipid peroxidation, PLC activation, calcium flux, and mtROS generation, which triggered NLRP3 inflammasome and caspase-1 activation, leading to IL-1β release and GSDMD-mediated cellular scorching in Kupffer cells (Figure 3). GO-L (large lateral size, 583 ± 343 nm) induced a significantly stronger IL-1β was significantly stronger than that of GO-S (small lateral size, 91 ± 79 nm) [73]. Small and large GOs vary from one another in their cellular uptake and membrane absorption, which probably causes their discrepancies. Ma et al. demonstrated that GO-L adsorbed more strongly on the plasma membrane and was less phagocytosed, as GO with larger lateral dimensions is more difficult to be absorbed [124]. One effect of plasma membrane uptake is that it led to a stronger interaction of GO with TLR4, which in turn activates the NF-κB pathway and results in stronger NLRP3 inflammasome activation after cellular uptake, accompanied by increased inflammatory cytokine production. GO sheets, on the contrary, are more likely to be taken up by cells due to their lack of TLR4 activation and reduced capacity to trigger NLRP3 inflammasome activation.

### 3.3. High Aspect Ratio of Nanorods and Nanowires

High aspect ratio NPs in the shape of nanotubes, nanowires, and nanoribbons have unique physical and chemical properties and are gradually being developed for applications such as titanium dioxide nanoribbons (TiO_2_-NB) as photocatalysts and MWCNTs in superconducting materials, optical devices and biomedical applications [24,125,126]. It has been shown that the length and aspect ratio of nanorods and nanowires are important for their role in different cells, and various nanoparticles with high aspect ratios exhibit aspect ratio-dependent toxicity in vitro and in vivo, including CeO_2_ nanorods, silicon, nickel, or silver nanowires, and alumina nanotubes [127]. Some materials with high aspect ratios, such as asbestos fibers, cannot be phagocytosed by macrophages when their length exceeds 15 μm, causing impaired cellular phagocytosis and chronic granulomatous inflammation of the corresponding tissues. Chronic granulomatous inflammation and fibrosis induced by ENMs is accompanied by activation of the NLRP3 inflammasome and production of IL-1β. At a critical length of 200 nm and an aspect ratio of 22, CeO_2_ nanorods form stacked bundles of up to 6–7 μm that could puncture cell membranes, resulting in the inability of macrophages to phagocytose. 1.1–2.9 μm stacking was also able to puncture lysosomes, leading to lysosomal damage and release of histone B, and ultimately NLRP3 inflammasome activation and IL-1β production [24]. TiO_2_ nanowires activated NLRP3 inflammasome in mice during the development of pulmonary fibrosis. It has been shown that immunostimulatory activity depends on their diameter, morphology, crystallization ability, hydroxyl concentration, and surface chemistry. Among them, the shape has been identified as a key factor for effective antigen uptake. The shape of aluminum nanorods adjuvants was able to activate NLRP3 inflammatory tissues and elicit corresponding antigen-specific immune responses, where morphology was the key factor of influence. Furthermore, aluminum hydroxide nanorods were more effective than nanoplates and nanopolyhedra in activating the NLRP3 inflammasome [128]. As the most redox-active substance, AlOOH nanorods with the highest hydroxyl content stimulated NLRP3 inflammasome and released IL-1β in THP-1 cells and BMDCs [129]. According to these results, nanorods of high aspect ratio play an inductive role in NLRP3 inflammasome activation and IL-1β production. The specific mechanism of induction is not unified, but the length and diameter variables of aspect ratio can be considered when exploring the induction effect.

### 3.4. Surface Silanol Density of Fumed Silica Nanoparticles

Studies have revealed that not all amorphous silica is the same and that the exceptional toxicity of fumed silica in contrast to colloidal silica is caused by its structure and surface chemistry, as well as its fused chain-like shape formed by rapid thermal quenching and high-temperature synthesis (>1300 °C). Fumed silica presents hazardous potential because of its high silanol density, siloxane ring structure, and “string-of-pearl” aggregate structure, which enables disruption of the membranes, produces ROS, and induces an inflammatory state, including activating the NLRP3 inflammasome [85,130,131].

Zhang et al. showed that the membrane destabilization and toxicity of fumed silica may be ascribed to particular surface features as a consequence of the reconstruction of strained three-membered rings (3MRs) and surface display of silanols groups [85]. The amount of electrostatic interaction between the fumed silica surface and plasma membrane phospholipids is determined by the density of surface silanol groups (≡Si-OH), which are partly deprotonated at physiological pH to create ≡Si-O− [85,132]. This may result in a compromise of the integrity of the plasma membrane, which may cause red blood cells to hemolyze [133,134]. Another effect of the cleavage of strained 3MRs at the silica surface is the production of hydroxyl radicals, which may exacerbate plasma membrane disruption and create a hazardous signal that triggers the NLRP3 inflammasome activation [135,136].

In contrast to other ENMs (rare earth oxides [40], graphene [98], carbon nanotubes [137], CeO_2_ [113], AlOOH [138], and Ag nanowires [64]), fumed silica shows a unique pathway by triggering NLRP3 inflammasome assembly, but not disturbing the lysosome’s function. Plasma membrane perturbation and potassium efflux both result from the change of the intracellular biological response after the fumed silica exposure to the plasma membrane [29,139]. Additionally, exposure to amorphous SiO_2_ NPs increased the production of ROS, activated the NLRP3 inflammasome, and up-regulated the expression of HMGB1, thereby triggering the TLR4/MyD88/NF-κB signaling pathway, which led to the inflammatory injury in HUVECs [46].

The acute pulmonary inflammation of fumed silica can be reduced by decreasing the density of bioactive surface silanols [92]. While calcination might achieve this effect, experimental rehydration methods such as submersion in an aqueous solution make it simple to restore these silanols. Doping Ti or Al to replace Si caused a perpetual reduction in silanol density and a lasting decrease in surface reactivity as an alternate way of reducing silanol density. Incremental dope of Ti and Al might lower surface silanol display and expression of 3MRs, which diminished hydroxyl radical formation, membrane disruption, K^+^ efflux, NLRP3 inflammasome activation, and toxicity in a dose-dependent manner in vitro. The suppression of NLRP3 inflammasome activation was also observed in BMDMs. In addition, doping with Ti and Al reduced acute pulmonary inflammation. It is important to note that since Ti and Al, as well as their oxide forms, possess minimal toxicity, doping is supposed to be safe by design approach [140]. All of these results suggest the possibility of using doped materials as a sustainable design strategy for situations when exposure to fumed silica might result in lung inhalation.

Sun et al. revealed that fumed silica has the potential for sub-chronic pulmonary effects following repeated doses [141]. Due to the rapid dissolution and elimination of fumed silica, instilling fumed silica in a single bolus dose of 21 mg/kg failed to cause prolonged IL-1β generation or sub-chronic lung damage. In contrast, the repetitive dosage delivery of 3 × 7 mg/kg fumed silica, spaced one week apart, constantly activated the NLRP3 inflammasome and might result in persistence and inflammation in the lung. At 3 × 3 mg/kg, repeated administration can promote collagen deposition. When utilizing titanium-doped fumed silica for repeated instillation, the sub-chronic pro-inflammatory effects vanished in a period.

### 3.5. Crystallinity, Aspect Ratio, and Length of Nanocellulose Materials

Nanocellulose (NC) is considered a reliable, green material and can be classified as cellulose nanocrystal (CNC), cellulose nanofibers (CNF), and bacterial nanocellulose (BNC), whose basic properties are high degradability, low density, and non-toxicity. Due to their distinct chemical morphology and attractive mechanical features, specific nanomaterial properties (e.g., high specific surface area and high aspect ratio), low toxicity, biodegradability, and biocompatibility, they are widely used in package materials, pollutant treatment, drug delivery, histological engineering, aerogels, transducers, pharmaceuticals, electronics, and other fields [142,143,144,145]. Although NC is considered to be biocompatible, many recent studies have shown that nanofibers, especially fibrin nanocrystals, can be dangerous and cause inflammatory responses after lung exposure, with similar responses on macrophage cell lines in vitro. Phagocytosis of shorter nanofiber samples (length = 280 nm CNC) by Kupffer cells induced mtROS production, caspase-3/7/1 activation, lysosomal damage, histone B release, NLRP3 inflammasomes assembly as well as IL-1β augmentation [146,147]. A recent report showed that both CNC and CNF can induce lysosomal damage, and longer length (200–300 nm) CNC had a lower activation threshold which was more likely to activate the NLRP3 inflammasome and induced IL-1β. In addition, the pro-inflammatory effect of CNC was associated with its higher crystallinity index, higher surface hydroxyl density, and ability to induce ROS production [147]. The crystallinity and surface reactive groups such as hydroxyl groups of nanoparticles reflect the intrinsic ability of nanomaterials to induce ROS generation, which was confirmed by the colorimetric fluorescence activity of dichlorofluorescein. The resultant changes in surface hydroxyl content and surface reactivity with material crystallinity determined the lysosomal damage [148]. Asbestos, high aspect ratios fiber metabolites such as fibrillar polypeptide amyloid beta, and monosodium urate crystals are known to activate the NLRP3 inflammasome, and various high aspect ratio nanomaterials can generate IL-1β in the THP-1 cell line [72,146]. However, it has also been reported that the most important parameter in the cytotoxic and inflammatory response of nanofiber-induced KCs is the length, not the aspect ratio [137].

### 3.6. Aggregation of 2D Molybdenum Disulfide

Due to their outstanding light-heat conversion, high electrochemical activity, carrier transport efficiency, and single- and two-photon fluorescence imaging capabilities, 2D molybdenum disulfide (MoS_2_) materials are rapidly being applied in the biomedical area. Although generally regarded as biocompatible, recent data have demonstrated that MoS_2_ may be dangerous in some biological cases. For instance, it has been shown that sequestration in the liver necessitates consideration of potential detrimental effects in this organ during drug carrier applications for MoS_2_ [149].

Yang et al. found that MoS_2_ quantum dots activated the NLRP3 inflammasome based on the transformation of the inactive precursor of caspase-1 into an active state and the cumulative generation of inflammatory markers, which led to microglia cell death by pyroptosis relied on caspase-1 [150].

Three aqueous suspended forms of MoS_2_, aggregated MoS_2_ (Agg-MoS_2_), MoS_2_ exfoliated by lithiation (Lit-MoS_2_), and MoS_2_ dispersed by Pluronic F87 (PF87-MoS_2_) were the subject of a thorough investigation by Wang et al. regarding their potential pulmonary hazards [138]. Compared to 2D-MoS_2_, including Lit- and PF87-MoS_2_, Agg-MoS_2_ greatly boosted the pro-inflammatory effects in the lung. Additionally, none of the 2D MoS_2_ produced lung sub-chronic effects. According to these findings, exfoliation reduces the toxicity of Agg-MoS_2_, which may prompt the safer implementation of 2D-MoS_2_ in biological engineering.

Furthermore, Li et al. assessed the effects of Agg-MoS_2_ and PF87-MoS_2_ on three major liver cell types including KUP5, LSECs, and Hepa 1-6 cells [151]. KCs were the only cell type in which MoS_2_ induced dose-dependent cytotoxicity. The effect of MoS_2_ might be attributed to the dissolution of nanosheets and the release of hexavalent Mo, which can cause ROS production and caspase 3/7-mediated apoptosis in KUP5 cells. A separate response pathway including lysosomal damage, NLRP3 inflammasome activation, caspase-1 activation, and proinflammatory interleukin generation was activated as a result of the phagocytosis of Agg-MoS_2_, without any signs of pyroptosis. It may be inferred that Mo release serves as a driving factor for the initiation of apoptosis since this response pathway is not activated by PF87-MoS_2_ or soluble Mo (VI). The lack of pyroptosis upon exposure to Agg-MoS_2_ may be caused by early caspase 3/7 activation, which results in gasdermin D cleavage at sites inhibiting the formation of caspase 1-induced pore-forming fragments [152]. These findings are consistent with the previous finding that V_2_O_5_ nanoparticles activate caspase 3 and 7, which prevents KUP5 cells from producing gasdermin pore-forming subunits or proceeding into pyroptosis [72]. In conclusion, Mo release and MoS_2_ dispersion both significantly affect the viability of the KCs. The Agg-MoS_2_ has distinct effects on cells, while the soluble one is essential for producing toxicity.

### 3.7. Autophagy Disruption by REOs

An important role in clearing active NLRP3 inflammasome complexes is played by the autophagy pathway, a homeostatic system that can affect the severity of inflammation. Autophagy induction, autophagosome synthesis, autophagosome fusion, and degradation in lysosomes are the three main stages of autophagy flux. Activated NLRP3 inflammasome complexes can form spontaneously or in response to stimuli that damage lysosomes in cells. Autophagosomes envelop these complexes, which are then transported to lysosomes through vesicle fusion. The subsequent degradation of inflammasomes by lysosomes assists to restore baseline IL-1β generation [153]. These mechanisms are susceptible to numerous factors. Rapamycin can counteract the effects of mTOR inhibition on autophagy induction, speeding up the autophagic flux [154]. PI3K activity is inhibited by 3-Methyladenine, which prevents the generation of autophagosomes. Chloroquine inhibits autophagosome fusion by interfering with lysosome acidification, which results in an accumulation of autophagosomes [155].

REOs can activate the NLRP3 inflammasome by disrupting autophagy. Mirshafiee et al. found that REOs activated the NLRP3 inflammasome, caspase 1, and pyroptosis in KCs. Knocking down the pore-forming protein gasdermin D reversed the pyroptosis condition including membrane blebbing, enhanced membrane permeability, cell swelling, and IL-1β release. Additionally, macrophage cell lines such as J774A.1, RAW 264.7 cells and BMDMs were found to be affected by the cytotoxic effects of REO NPs. These phagocytic cell types further displayed pyroptotic characteristics and elevated IL-1β production [41].

Li et al. compared the function of autophagy in modulating inflammasome activation and IL-1β level between MWCNTs and REO NPs using the THP-1 cell line and BMDMs. In BMDMs and THP-1 cells, REO NPs were more effective stimulators of IL-1β secretion than MWCNTs. While MWCNTs induced autophagosome fusion in cells to dramatically eliminate the bioactivated NLRP3 complexes, REO NPs hindered autophagosome fusion within lysosomes after transformation into urchin-shaped structures in lysosomes. As a result, the NLRP3 inflammasome accumulated and continued to produce IL-1β. Treatment with REO NPs might result in a significant amount of lysosomal protein dephosphorylation as compared to extracts obtained from untreated cells. It has been shown that the removal of the phosphate group treated with REOs and the deficiency of the phosphate group from 12–25% of the phosphopeptides by the REO treatment both impaired the β -galactosidase activity. Additionally, lysosomal pH was measured using cellular staining with fluorescent dye. The fluorescence intensity of the dye decreased in NPs treatment group, indicating that REO NPs intervene in lysosome acidification [156]. Collectively, the primary impact of REOs is autophagy inhibition, which disturbs the homeostasis of activated NLRP3 inflammasome. REOs turn into RE-phosphate with a sea urchin structure after being internalized into lysosomes. The drop in enzymatic activity of lysosomes owing to the lysosomal dephosphorylation and alkaline change is a sign of lysosomal dysfunction induced by this biotransformation. Lysosomal dysfunction causes inhibition and accumulation of autophagosome as well as a futile degradation of activated NLRP3 inflammasome. Excessive IL-1β production results from the disruption of NLRP3 inflammasome homeostasis.

### 3.8. Na^+^/K^+^ ATPase Inhibition by V_2_O_5_

By hydrolyzing ATP and pumping 3 Na^+^ out of the cell in return for 2 K^+^ entering the cell, Na^+^/K^+^ ATPase enables mammalian cells to main a high ratio of K^+^-to-Na^+^ [157]. Vanadium ions (V^5+^) can block the Na^+^/K^+^ ATPase in the cell surface membrane, which can lead to Na^+^ release from the surface membrane and a drop in cellular K^+^ levels [158]. Wang et al. evaluated the Na^+^/K^+^ ATPase activity in KUP5 cells exposed to V^5+^ and V_2_O_5_ NPs [72]. The Na^+^/K^+^ ATPase activity was shown to be inhibited by both the particles and the V^5+^, which decreased cell viability. When KUP5 cells were treated with V^5+^ or V_2_O_5_ NPs, the intracellular K^+^ levels decreased significantly in a time-dependent manner. As demonstrated by the results above, V_2_O_5_ NPs can activate NLRP3 by disrupting the Na^+^/K^+^ ATPase and causing potassium leakage in KUP5 cells. Na^+^/K^+^ ATPase inhibition results in a cessation of intracellular K^+^ pumping, which lowers intracellular potassium levels as a consequence of K^+^ efflux through leak channels. NLRP3 inflammasome assembly, caspase 1 activation and the generation of IL-1β can all be triggered by potassium efflux. Despite the activation of NLRP3 and caspase 1, V_2_O_5_ NPs failed to induce pyroptosis in KUP5 cells. One possibility is that the inactive pore-forming GSDMD fragments produced by caspase 1 was late in onset, allowing early caspases 3 and 7 activation to induce apoptosis [152]. Table 2 summarizes the property-activity relationships for ENMs.

## 4. Conclusions

In this review, we have summarized the pathways of ENMs-induced activation of NLRP3 inflammasome. TLR4 on the cell membrane recognizes damage-associated molecular patterns, which activates NF-κB and induces the production of pro-IL-1β and NLRP3. The perturbation of the plasma membrane is determined by interactions at the nano-bio interface, and ROS is generated by NADPH oxidase activation. Mitochondria represent another significant generator of ROS in the cells. The lysosome destabilization and cathepsin B release may result from excessive ROS generation, which triggers the inflammasome activation cascade. In addition, potassium efflux induced by NPs elicits a high level of ROS, leading to the activation of NLRP3 inflammasome. Furthermore, the NLRP3 activation may be influenced by the surface reactivity of CNTs, the lateral size of GO, the high aspect ratio of nanowires, surface silanol density of fumed silica NPs, physical characteristics of nanocellulose materials, aggregation of 2D molybdenum disulfide, disruption of autophagy by REOs, and inhibition of Na^+^/K^+^ ATPase by V_2_O_5_. Although many researchers have attempted to explore the characteristics of ENMs one at a time in each study, changes in one aspect might lead to differences in the others, making it difficult to pinpoint which characteristics most significantly impact ENMs’ bioactivity. It is considerably challenging to manage the properties of ENMs in practical applications. As a result, it is essential to create libraries of well-synthesized ENMs with distinct features such as composition, diameter, shape, charge, crystallinity, aggregation state, aspect ratio, and surface modification, which enable us to associate the integrated effects of all related characteristics of a certain ENMs with NLRP3 inflammasome activation. Furthermore, there may be other ENM-induced pathways that result in NLRP3 inflammasome activation, hence more studies are warranted to understand the underlying relationship between NLRP3 and ENMs. To gain new insights into the toxicological mechanisms of ENMs in an unbiased way, recent technological advances including omic approaches such as RNA sequencing, proteomics, and metabolomics provide opportunities to comprehensively understand the new pathways of nanotoxicity, cellular phenotypes induced by ENMs, and interactions between NPs and human organs. The findings facilitate the mitigation of toxicological responses induced by ENMs via signaling pathway prevention or innovative customization by inhibiting the predominant cytotoxic effect of ENMs. Different methods for designing ENMs that are less hazardous to biological systems and the environment include metal doping, surface coating, oxidation state modification, and aspect ratio adjustment. These methods target different mechanisms of nanotoxicity by modifying the physicochemical characteristics of ENMs, which ameliorate their toxic effects. However, under some exposure conditions and circumstances, the safer designed NPs may be ineffective for certain biomedical applications. Further appraisal of these nanomaterials will be needed to define the new and valid borderlines. With the expanding amount of novel ENMs and their widespread use, it is crucial to gain a more in-depth understanding of nano-NLRP3 inflammasome interactions with advanced technologies and create safer design concepts of ENMs for human-friendly nanotechnology and nanoproducts.

## Figures and Tables

**Table 1:** Mechanisms involved in NLRP3 inflammasome activation by ENMs.

ENMs	Characterization	Model	Cellular Responses/Effects	Ref.
MWCNT	Long tangled CNT with OD 8–15 nm and length 10–50 µm; Long rigid CNT with OD < 50 nm and length ~ 13 µm	Human monocyte-derived macrophages	Secretion of inflammation-related proteins including lysosomal proteins	[34]
MWCNT	MWCNT with diameter 110~200 nm	THP-1 cells	Lysosomes destabilization, cathepsin B release and NLRP3 inflammasome activation	[35]
MWCNT	Needle-like MWCNTs with diameter 50 nm; thick MWCNTs with diameter 150 nm	Fischer 344/Brown Norway F1 hybrids rats, MeT5A and RAW264.7 cells	Lysosome damage, cathepsin B release, and more inflammogenicity induced by needle-like MWCNTs	[37]
MWCNT	AP-MWCNT, PD-MWCNT and COOH-MWCNT with diameter 20~30 nm	C57BL/6 mice and THP-1 cells	Inhibition of lysosomal damage, cathepsin B release, NLRP3 inflammasome activation and IL-1β production induced by PF108-dispersed tubes	[22]
MWCNTs, SWCNTs	Long and slender MWCNTs with size 12 nm × 10 μm; intermediate MWCNTs with size 10 nm × 3–6 μm; short and slender MWCNTs with size 9.5 nm × 1 μm; short and rigid MWCNTs with size 49 nm × 3.86 μm; long SWCNTs with size 0.7–1.1 nm × 0.3–2.3 μm; short SWCNTs with size 1–2 nm × 0.5–2 μm	J774A.1 and WI38-VA13 cells	ROS generation, NLRP3 activation, IL-1β production, and macrophage-guided myofibroblast transformation	[50]
MWCNTs, SWCNTs	MWCNTs with average diameters 20–30 nm and lengths 10–30 μm; SWCNTs with average diameters 0.8–1.2 nm and lengths 1–5 μm	C57BL/6 mice, THP-1 cells, and BMDMs	Lysosomal damage, oxidative stress and subsequent NLRP3 inflammasome activation through NADPH oxidase activation	[64]
Graphene platelet(GP)	GP1 with lateral size 80~300 nm; GP2 with lateral size 250~400 nm	THP-1 cells	Lysosomes destabilization, cathepsin B release and NLRP3 inflammasome activation	[35]
GO	Small GO with lateral size 50–300 nm and large GO with lateral size 10–40 µm	HMDM, HEK 293, and THP-1 cells	Cellular uptake of GO, cathepsin B release, K^+^ efflux and NLRP3 inflammasome activation	[31]
GO	Size 50~200 nm	BALB/c mice	ROS production, TLR-4 activation, macrophage polarization, IL-1β and TNF-α production, NF-κB activation, and initiated IL-6 expression	[44]
GO	GO-S with lateral size 97 ± 79 nm, height 1.3 ± 0.9 nm GO-L with lateral size 583 ± 343 nm, height 1.2 ± 0.5 nm	KUP5, LSEC, and Hepa 1-6 cells	Plasma membrane lipid peroxidation, calcium flux, mtROS generation, and NLRP3 inflammasome activation	[73]
REO	Y_2_O_3_, La_2_O_3_, CeO_2_, Eu_2_O_3_, Gd_2_O3, Dy_2_O_3_, and Yb_2_O_3_ with primary size 18–60 nm; Nd_2_O_3_, Sm_2_O_3_ and Er_2_O_3_ with primary size 100–140 nm	THP-1 cells	Membrane strip, nanoparticle biotransformation into urchin shaped structures, cathepsin B release, NLRP3 inflammasome activation, IL-1β release, TGF-β1 and PDGF-AA production induced by the released RE ions	[40]
REO	Primary size 10 to 140 nm	KUP5, J774A, RAW 264.7, Hepa 1-6 cells and BMDMs	Lysosomal damage, NLRP3 inflammasome activation, caspase 1 activation, and pyroptosis in KCs and other phagocytic cell types but not in Hepa 1-6 cells	[41]
CeO_2_	Relatively constant diameter 6–10 nm but different lengths above 33.2 nm	THP-1 cells	Lysosomal damage, cathepsin B release, and IL-1 production when CeO_2_ with length ≤ 200 nm and aspect ratio ≤ 22 nm	[24]
LNs	Upconversion nanocrystals with diameter approximately 25 nm; Y_2_O_3_ with size from dozens of nanometers to several microns; Nd_2_O_3_ with size about 500 nm	C57BL/6 mice, L929 cells and THP-1 cells	Suppressed ROS generation (largely from NADPH oxidase) and decreased NLRP3 inflammasome activation induced by LNs with RE-1 coating	[81]
Au	Mean diameters 4.5, 13, 30 and 70 nm	C57BL/6 mice and BMDCs	ROS generation, proteasomal degradation by targetting autophagy protein-LC3 (microtubule-associated protein 1-light chain 3) and NLRP3 inflammasome activation induced by ultrasmall size Au NPs(4.5 nm)	[61]
Ag	Ag wires with average diameters 65 nm and lengths 20 μm	THP-1 cells, and BMDMs	Lysosomal damage, oxidative stress and subsequent NLRP3 inflammasome activation through NADPH oxidase activation	[64]
Ag	Size 199.40 ± 21.45 nm	Giant and large unilamellar vesicles	Invaginations and intraluminal vesicles, increased membrane tension, and membrane perforation	[83]
Cobalt	Mean diameter 20 nm	L02 cells	MtROS generation, and NLRP3 inflammasome activation	[75]
CuO	Size 50 nm	J774A cells	Pro-IL-1β production, the MyD88-dependent TLR4 signaling pathway activation and NF-κB activation	[45]
IONPs	Size approximately 25 nm	C57BL/6 mice, THP-1 cells, and BMDMs	ROS generation from NADPH oxidase and NLRP3 inflammasome activation	[65]
TiO_2_	Long fiber-shaped titanium dioxide nanobelts with diameter 60–300 nm and length 15–30 µm; short nanobelts with diameter 60–300 nm and length 0.8–4 µm; spherical nanobelts with diameter 60–200 nm	C57BL/6 mice and primary murine alveolar macrophages	Lysosomal damage and cathepsin B release induced by long fiber-shaped titanium dioxide nanobelts but not spherical nanobelts	[23]
TiO_2_	Size 5~6 nm	Neuroinflammation in the mouse hippocampus	TLR4 expression, activation of NF-κB-inducible kinase (NIK), IKKs and IkB phosphorylation, ubiquitination and degradation of IkB, and NF-κB activation	[42]
TiO_2_	Primary size 20–80 nm	C57BL/6 mice and BMDCs	ROS generation and NLRP3 inflammasome	[58]
TiO_2_	50 μg/m^3^	BALB/c mice	Increased ROS levels, promoted expression of IL-1β, IL-18, NLRP3 activation, exacerbated airway inflammation and hyperresponsiveness	[59]
TiO_2_	Size 419 ± 83 nm	Macrophages and dendritic cells	Mechanosensitive channel-mediated K^+^ efflux, activation of inflammasomes in a caspase-1 and NLRP3-dependent manner	[93]
Zinc oxide	Size 41.7 ± 26.3 nm	A549 cells	NLRP3 inflammasome activation via a ROS-NF-κB-NLRP3 signaling pathway	[70]
Zinc oxide	Mean diameter approximately 35.65 ± 7.93 nm	SKH-1 hairless mice and HaCaT cells	Mitochondrial dysfunction, mtROS generation, and NLRP3 inflammasome activation	[71]
Nano silica	Size 70 nm	C57BL/6J mice	ROS generation, increased NLRP3 inflammasome activity, and placental inflammation	[49]
Nano silica	Median diameter 1.6 μm	J774A.1 and WI38-VA13 cells	ROS generation, NLRP3 activation, IL-1β production, and macrophage-guided myofibroblast transformation	[50]
Nano silica	Mean size 50.16 ±5.55 nm	Fischer 344 rats and AC16 cells	ROS generation, the NLRP3/Caspase-1/GSDMD signaling pathway activation, pyroptosis and cardiac hypertrophy	[66]
Nano silica	Size 20 nm	Macrophages	ROS production, plasma membrane perturbation, decreased membrane fluidity, increased intracellular [Ca^2+^]i	[84]
MSNs	Size 30~70 nm	Wistar rats	Up-regulated expression of TLR4, MyD88, NF-κB, p65, and caspase-3, and increased serum pro-inflammatory cytokines	[47]
MSNs	Mean diameter approximately 109.2 nm	BALB/c mice and hepatic L02 cells	ROS generation, NLRP3 inflammasome activation, caspase-1-dependent pyroptosis, promoted liver inflammation and hepatocyte pyroptosis	[54]
MSNs	Average size approximately 75 nm	Caco-2 cells	ROS generation, NLRP3 inflammasome overactivation, inhibited expression of the autophagy proteins LC3-II and Beclin1, and decreased intestinal inflammation	[55]
Amorphous silica NPs	Size 15 nm	HUVECs	Lysine acetylation, TLR4 binding, the MyD88 and NF-κB signaling pathways activation	[46]
Amorphous silica NPs	Diameter 30–1000 nm	C57BL/6 mice and THP-1 cells	ROS generation, NLRP3 inflammasome activation and IL-1β production induced unmodified microsized 1000-nm SP(mSP1000). IL-1β inhibition by decreasing ROS generation induced by the surface modification(eCOOH, eNH2, eSO3H, eCHO) of mSP1000	[25]
Fumed SiO_2_	Size 65~191 nm	THP-1 cells	Plasma membrane perturbation, hydrogen-bonded silanol groups formation, potassium efflux, NLRP3 inflammatory vesicle activation, and IL-1β production	[87]
Fumed SiO_2_	Size 16 nm	BEAS2B, RAW 264.7 and THP-1 cells	Hydrogen bonding and electrostatic interactions, IL-1β secretion, plasma membrane perturbation and ROS production	[85,86]
Fumed silica	Size 16 ± 2 nm	THP-1 cells	K^+^ efflux, aggregation of NLRP3, ASC, and caspase-1 subunits of inflammasomes	[92]
PSNPs	Diameter 100 nm	C57BL/6 mice, AML12, Hepa1–6, and HepG2 cells	Cell membranes ruptured, ROS generation, NLRP3 pathway activation and neutrophil extracellular traps formation	[56]
PSNPs	Diameter about 102 nm	C57BL/6 mice, NCM460 cells	Increased oxidative stress, ROS generation, NF-κB/NLRP3 pathway activation, promoted expression of inflammatory factors (TNF-α, IL-6, and IFN-γ), deteriorated inflammation and increasing permeability in mice duodenum	[57]
PSNPs	Diameter ∼100 nm	Human macrophages	Mitochondrial damage, ROS generation, TXNIP release, NLRP3 activation and IL-1β release induced by amino-functionalized polystyrene nanoparticles	[79]
CdSe/ZnS quantum dots	Diameter approximately 7.1 nm	C57BL/6 mice and L02 cells	MtROS generation, NLRP3 inflammasome activation, hepatocyte pyroptosis, liver inflammation and dysfunction.	[77]
Ag_2_Se quantum dots	Average size 2.8 ± 0.5 nm	ICR mice and BV2 microglial cells	ROS generation (particularly mtROS), NF-κB activation and NLRP3 inflammasome activation, pro-IL-1β upregulation and IL-1β release	[78]
Nano diamonds	Average size 100 nm	THP-1 cells	Lysosomes destabilization, cathepsin B release and NLRP3 inflammasome activation	[30]
CBNPs	Hydrodynamic size 200–400 nm in aqueous suspension	Sorague-Dawley rats and 16HBE cells	ROS generation, decreased miR-96, increased FOXO3a and NLRP3 inflammasome activation	[60]
CNC-AEMA2	Diameter 10–20 nm and length 100–200 nm	J774A.1 cells	MtROS generation, and NLRP3 inflammasome activation	[74]
AAHPs	Size 20~35 nm	THP-1 cells	Plasma membrane perturbation, potassium efflux, lysosomal damage, mitochondrial dysfunction, mtROS production and IL-1β production	[88]
PDLA- CS, PEG-PLGA-PLL, and PEG-PS/CaP NPs	Size 100 nm	BEL-7402 and LO2 cells	K^+^ efflux, escaped lysosomes, calcium ion concentration disruption, and binding of caspase-9, AFP-1, and cytochrome c	[91]
Gd@C_82_(OH)_22_	Size 20~100 nm	MyD88/TLR2/TLR4 knockout C57/BL mice	K^+^ efflux, and NLRP3 inflammasome activation	[94]

**Table 2:** Property activity relationships for ENMs.

Property		ENMs	Characterization	Model	Cellular Responses/Effects	Ref.
**Surface reactivity**	Length	MWCNTs	NTlong1 with mean length 13 μm; NTlong2 with mean length 36 μm	THP-1 cells and Met5a cells	Raised the levels of IL-1β and IL-6 induced by NTlong1 and NTlong2, whereas elevated IL-8 expression induced by NTlong1 by itself	[103]
		MWCNTs	Diameter 20–100 nm and length approximately 50 μm	THP-1 cells	Increased bioactivity and the most IL-1β release induced by long and rigid MWCNTs with a needle-like appearance or appearing as individual fibers	[104]
		MWCNTs	Long tangled CNT with OD 8–15 nm, and length 10–50 µm; long rigid CNT with OD < 50nm, and length ~ 13 µm	Human monocyte-derived macrophages	Higher cytotoxicity, increased IL-1α and IL-1β generation, and stimulated generation of lysosomal and inflammatory proteins induced by long, needle-like MWCNTs	[34]
		MWCNTs	Short MWCNT with OD 5–20 nm, and length 1 -> 10μm; long tangled MWCNT with OD 8–15 nm, and length 10–50 μm; long needle-like MWCNT with OD > 50 μm, and length ~ 13μm	Human primary macrophages	NLRP3 inflammasome activation via ROS production, cathepsin B release, P2X7 receptor, and Src and Syk tyrosine kinases induced by long, needle-like MWCNTs	[36]
	Suspension state	MWCNTs	Average diameter 20–30 nm and average length 10–30 μm	BEAS-2B, THP-1 cells, and alveolar macrophages	More prominent TGF-β1 and IL-1β production than non-dispersed tubes induced by well-dispersed AP- and PD-MWCNTs induced	[39]
	Charges	MWCNTs	Median diameter of 44 nm	C57BL/6 mice	Reduced NLRP3 inflammasome activation and pulmonary toxicity induced by MWCNT with -COOH group	[110]
		CNTs, CNOs	p-CNTs with mean height 1.0 nm and mean length 325 nm; p-CNOs with mean size 6.2 nm	C57BL/6 mice and BMDCs	Reduced NLRP3 inflammasome activation and IL-1β production induced by CNTs and CNOs with benzoic acid functionalization	[111]
		MWCNTs	Hydrodynamic size in water: COOH, PEG, PEI-MWCNTs 140–290 nm; AP, NH2 and sw-NH2 MWCNTs 1900–2300 nm	BEAS-2B and THP-1 cells	Reduced NLRP3 inflammasome activation and IL-1β, TGF-1, and PDGF-AA production induced by MWCNT with anionic functionalization (COOH- and PEG-); intermediary effects induced by MWCNT with neutral and weak cationic functionalization (NH2 and sw-NH2); most potent biological effects induced by MWCNT with heavily cationic functionalization (PEI-)	[112]
	Surface coating	MWCNTs	Average hydrodynamic diameter 280 ± 36 nm(sonicated) or 406 ± 55 nm(unsonicated) and 0.5–40 mm in length	THP1 cells	Increased IL-1β secretion but decreased IL-6, TNF-α, and osteopontin production induced by Al_2_O_3_-coated MWCNTs	[115]
		MWCNTs	Diameter: PMWCNT 13.5 ± 1.5 nm; TMWCNT 7.5 ± 2.5 nm	C57BL/6 mice and BALF cells	More severe acute inflammatory effect than pristine MWCNT induced by acid-treated MWCNT	[116]
		SWCNT, graphene	Diameter: PF108-Hipco 0.7–1.1 nm; PF108-Arc 1.2–2.0 nm; PF108-SG65 0.7–1.0 nm; PF108-G 45 nm	BEAS-2B and THP-1 cells	Reduced NLRP3 inflammasome activation, and IL-1β production induced by PF108 coating SWCNT and graphene	[98]
Lateral size		GO	Size < 100 nm	MGC-803 and MCF-7 cells	Low cytotoxicity and size consistency	[122]
		GO	size 350 nm	PMØ, J774A.1, LLC, MCF-7, HepG2, and HUVEC cells	Better biocompatibility	[123]
		GO	Lateral size: GO-S 91 ± 79 nm; GO-L 583 ± 343 nm;	Kupffer cells, LSECs and hepatocytes	Plasma membrane lipid peroxidation, PLC activation, calcium flux, mtROS generation, NLRP3 inflammasome activation, caspase-1 activation, IL-1β production and cellular scorching	[73]
		GO	Size: GO-S 50 to 350 nm; GO-L 750 to 1300 nm	BALB/c mice and J774A.1, THP-1, HEK293, MEL, HUT102, RAMOS, and HepG2 cells	Stronger adsorption onto the plasma membrane with less phagocytosis, increased interaction with TLR4, NF-κB pathways activation, and NLRP3 inflammasome activation induced by larger GO	[124]
High aspect ratio		CeO_2_	Critical length of 200 nm and aspect ratio of 22	THP-1 cells	Lysosome damage, cathepsin B release, NLRP3 inflammasome activation and IL-1β production	[24]
		TiO_2_	6–12 μm in length, 60–140 nm in width, median aspect ratio ~ 80	BALB/c mice, and J774A.1 cells	NLRP3 inflammasome activation and pulmonary fibrosis	[128]
		AlOOH	average diameter of ∼20 nm and lengths of 150~200 nm.	THP-1 cells, and BMDCs	NLRP3 inflammasome activation and IL-1β production	[129]
Surface silanol density		Fumed silica	Diameter about 16 nm	BEAS-2B, RAW 264.7 and THP-1 cells	ROS generation, membrane perturbations, NLRP3 inflammasome activation, and IL-1β production	[85]
		Amorphous SiO_2_	Primary size 15, 30, 100 nm	HUVECs	ROS production, NLRP3 inflammasome activation, increased expression of HMGB1, TLR4/MyD88/NF-κB signal activation and inflammatory injury	[46]
		Fumed silica	Primary size 16 ± 2 nm	THP-1 cells and BMDMs	Reduced potassium efflux, NLRP3 inflammasome activation, and acute pulmonary inflammation induced by Ti and Al doping fumed silica	[92]
		Fumed silica	Primary size 16 nm	C57BL/6 mice and THP-1 cells	Fumed silica biopersistence, sustained macrophage recruitment and NLRP3 inflammasome activation induced by repetitive dosing of fumed silica	[141]
Crystallinity, aspect ratio and length		Cellulose nanocrysta (CNC)	Size 200–300 nm	KUP5 cells	MtROS generation, caspase-3/7 activation, apoptosis, lysosomal damage, cathepsin B release, NLRP3 inflammatory vesicles and caspase-1 activation, and IL-1β production	[146]
		Ag, CuO, and ZnO	Size: Ag 20 nm, CuO 60 nm, ZnO 50 nm	KUP5 and Hepa 1–6 cells	Caspase-3-induced apoptosis, MtROS generation	[72]
		SiO_2_	Size 20 nm	KUP5 and Hepa 1–6 cells	K^+^ efflux, caspase-1 activation, NLRP3 inflammasome assembly, IL-1β production, cleavage of gasdermin-D and pyroptosis	
Aggregation		Agg- MoS_2_	d_H_ 1334.8 ± 47.8 nm in water	C57BL/6 mice, BEAS-2B and THP-1 cells	More acute pro-inflammatory effects including IL-8, TNF-α, and IL-1β production.	[138]
		Agg- MoS_2_	Hydrodynamic size 730.9 ± 36.3 nm in water	KUP5, LSEC, and Hepa 1−6 cells	Lysosomal damage, NLRP3 inflammasome activation, caspase-1 activation, IL-1β and IL-18 production	[151]
Autophagy disruption		REOs	Primary size 10–140 nm, (except Cr2O3 ~190 nm)	KUP5, J774A.1, Raw 264.7 cells and BMDMs	Lysosomal damage, NLRP3 inflammasome activation, caspase-1 activation, pyroptosis, and IL-1β production	[41]
		REOs	Primary Size: La_2_O_3_ 26 ± 7 nm; Gd_2_O_3_ 47 ± 10 nm; Sm_2_O_3_ 186 ± 34nm; Yb_2_O_3_ 71 ± 13 nm	THP-1 and BMDMs	NLRP3 inflammasome activation, IL-1β production and interference in autophagic flux	[158]
Na^+^/K^+^ ATPase inhibition		V_2_O_5_	Primary size 395.0 ± 230.5 nm	KUP5 cells	Na^+^/K^+^ ATPase disruption, K^+^ leakage, NLRP3 inflammasome activation, IL-1β release and delayed caspase-1 activation	[72]

## Data Availability

Data presented in this manuscript is available from the corresponding author upon reasonable requests.
